# Encountering A Rare Entity:A Case Report of Prostatic Sarcomatoid Adenocarcinoma

**DOI:** 10.5146/tjpath.2025.14116

**Published:** 2026-09-15

**Authors:** Harshinee s, Renuka Patıl, Altaf Khan

**Affiliations:** Department of Pathology, Yenepoya Medical College, Yenepoya University, Karnataka, İndia; Department of Urology, Yenepoya Medical College, Yenepoya University, Karnataka, İndia

**Keywords:** Prostate, Sarcoma, Carcinoma, Biphasic, Rare

## Abstract

Sarcomatoid carcinoma of the prostate is a rare and aggressive malignancy characterized by biphasic differentiation with epithelial and mesenchymal components. Diagnosing this entity is challenging due to its overlapping histological features with other prostatic neoplasms. We report a case of an elderly patient who presented with acute urinary retention and a history of lower urinary tract symptoms, including difficulty in micturition and poor urinary stream. A transrectal ultrasound-guided biopsy was performed for an ill-defined lesion in the right lobe of the prostate with an elevated PSA level of 30 ng/mL. A bone scan revealed metastatic lesions. Histopathological examination confirmed sarcomatoid carcinoma with a Gleason score of 8 (Grade Group 4). The tumor exhibited a biphasic morphology, with malignant spindle cells interspersed with a high-grade carcinoma component. Immunohistochemistry demonstrated vimentin, smooth muscle actin (SMA), and SATB2 positivity in the sarcomatoid component, while the carcinomatous component showed positivity for pan-cytokeratin (pan-CK), CK7, and CK20. PSA was negative in both components. This case highlights the aggressive nature of sarcomatoid carcinoma and highlights the essential role of histopathology and immunohistochemistry in its accurate diagnosis and differentiation from other spindle cell tumors.

## Introduction

Sarcomatoid carcinoma of the prostate is a rare and aggressive malignancy, accounting for less than 0.1% of all prostate cancers (1). It is characterized by the presence of both carcinoma and sarcomatoid components, making it a significant histological challenge (2). In 2015, Markowski et al. reviewed a series of cases from Johns Hopkins Hospital, emphasizing its poor prognosis and limited treatment options (2). Other studies have reported its highly aggressive nature, frequent misdiagnosis, and the need for comprehensive immunohistochemical analysis for confirmation (2). The tumor is not well-defined in major classifications, making it difficult to standardize treatment protocols (3). Here, we present a case of sarcomatoid carcinoma of the prostate diagnosed initially a year ago, which is now being reported to highlight its pathological features. All clinical details were retrospectively obtained from the histopathology request form, with no additional patient interaction or further investigations. We also explore its clinicopathological and immunohistochemical characteristics while reviewing relevant literature to enhance our understanding of this rare entity.

## Case Report

An elderly patient presented with acute urinary retention and a history of progressive lower urinary tract symptoms, including difficulty in micturition and a weak urinary stream. Digital rectal examination (DRE) findings noted a firm Grade 4 prostate with no palpable nodules. Laboratory evaluation documented a markedly elevated prostate-specific antigen (PSA) level of 30 ng/mL. Ultrasonography described an enlarged prostate with a hetero-echoic echotexture and focal calcifications in the right lobe. A bone scan also revealed metastatic lesions.

**Figure 1:**
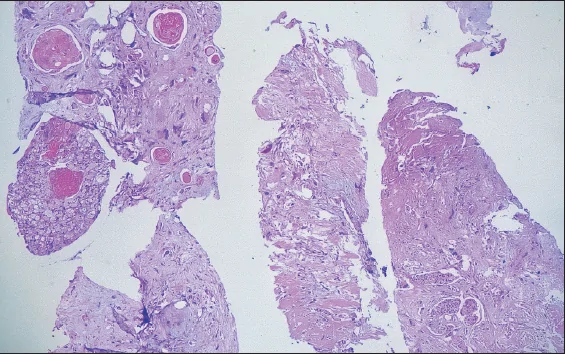
Hematoxylin and Eosin (H&E) Staining: shows a biphasic tumor pattern with carcinomatous glands and spindleshaped sarcomatoid tumor cells admixed with necrosis (x10).

**Figure 2:**
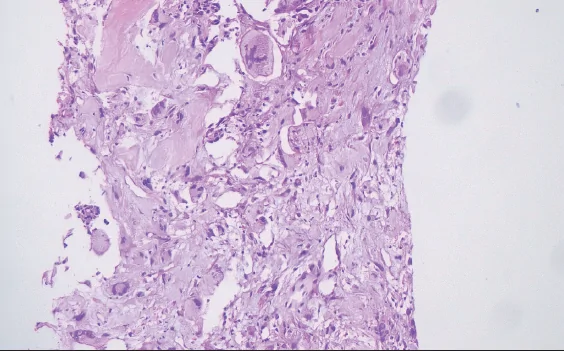
Hematoxylin and Eosin (H&E) Staining: shows the sarcomatoid component of the tumor composed of spindleshaped tumor cells exhibiting marked pleomorphism (x20).

Histopathological examination of retrieved slides from a previously performed transrectal ultrasound-guided biopsy (TRUS) confirmed the diagnosis of sarcomatoid carcinoma. (Gleason score 4+4=8, Grade Group 4). Microscopic evaluation with haematoxylin and eosin staining ( Figure 1,Figure 2) revealed a biphasic neoplasm with high-grade carcinomatous areas interspersed with sarcomatoid spindle cell proliferation, exhibiting marked nuclear atypia and pleomorphism (Figure 4). The absence of glandular differentiation within the sarcomatoid component further supported the diagnosis.

**Figure 3:**
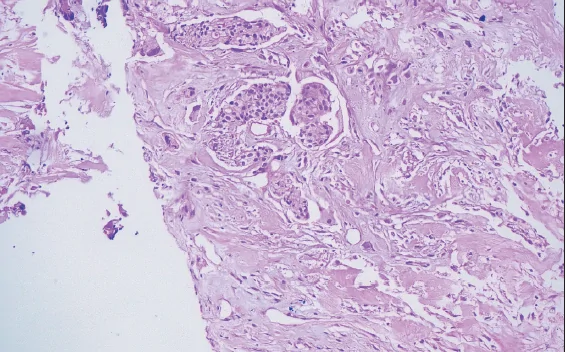
Representative images of SMA Immunohistochemical (IHC) Staining – shows membranous and cytoplasmic positivity in sarcomatoid component of tumor cells and is negative in carcinomatous component (x40). SMA: Smooth muscle actin

**Figure 4:**
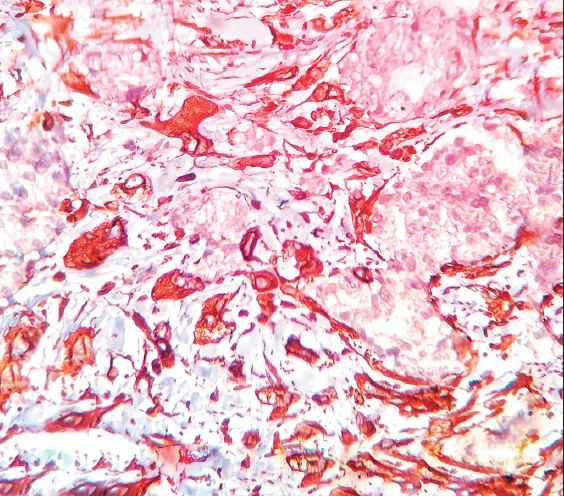
Representative images of SMA Immunohistochemical (IHC) Staining – shows membranous and cytoplasmic positivity in sarcomatoid component of tumor cells and is negative in carcinomatous component (x40). SMA: Smooth muscle actin

**Figure 5:**
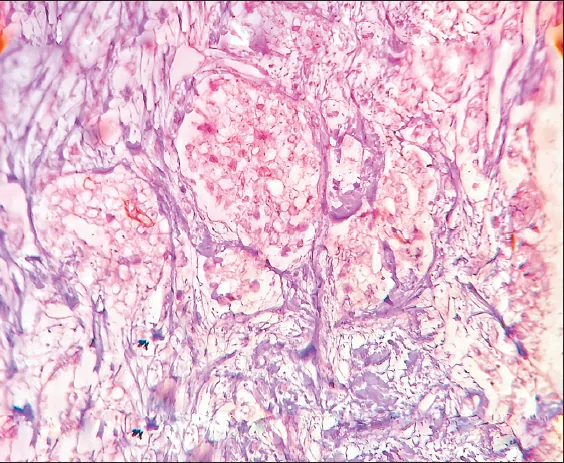
Hematoxylin and Eosin (H&E) Staining: shows the carcinomatous component of the tumor composed of malignant epithelial cells arranged in irregular glands and solid nests, displaying mild nuclear atypia (x20).

**Figure 6:**
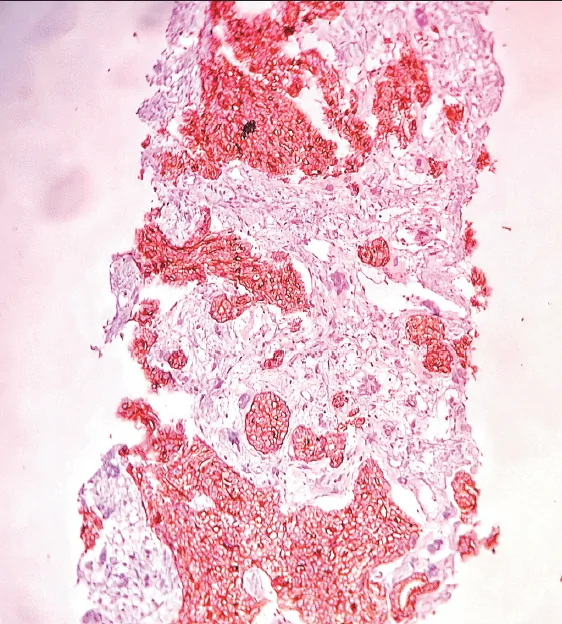
Representative images of Pan-CK Immunohistochemical (IHC) Staining- Carcinomatous component shows strong cytoplasmic and membrane positivity and is negative in sarcomatoid component (x20). Pan-CK: Pan-cytokeratin

**Figure 7:**
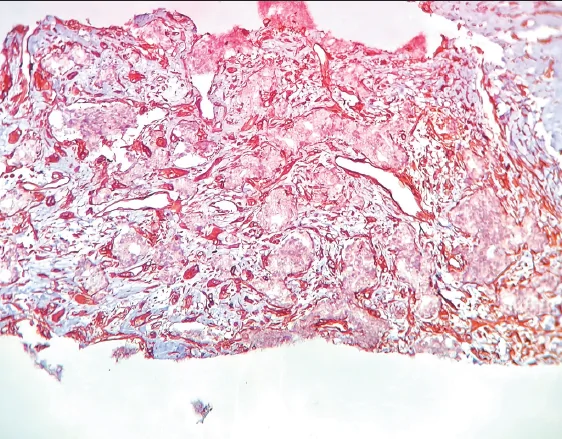
Representative images of Vimentin Immunohistochemical (IHC) Staining: Strong cytoplasmic positivity noted in sarcomatoid component, while carcinomatous component is negative (x20).

Immunohistochemical (IHC) stains, also retrieved from archival records, were helpful in differentiating the tumor components. The sarcomatoid areas showed strong positivity for vimentin and smooth muscle actin (SMA) (Figure 3), along with special AT-rich sequence-binding protein 2 (SATB2),(Figure 5) a marker indicative of mesenchymal differentiation. The carcinomatous component demonstrated retained epithelial markers, with positivity for pan-cytokeratin (Pan-CK) (Figure 6), cytokeratin 7 (CK7) (Figure 3), and cytokeratin 20 (CK20) (Figure 7). Notably, PSA expression was absent in both tumor components (Table 1), indicating dedifferentiation, a characteristic often associated with aggressive behavior in sarcomatoid prostate carcinoma.

**Table 1:** Immunohistochemical expression in sarcomatoid and carcinomatous components

**Components**	**Markers**	**Results**
**Sarcomatoid component**	Vimentin	Positive
	SMA	Positive
	SATB2	Positive
	PSA	Negative
**Carcinomatous component**	Pan-CK	Positive
	CK7	Positive
	CK20	Positive
	PSA	Negative

**SMA:** Smooth muscle actin, **SATB2:** Special AT-rich sequence-binding protein 2, **PSA:** Prostate-specific antigen, **Pan-CK:** Pan-cytokeratin, **CK-7:** Cytokeratin 7, **CK-20:** Cytokeratin 20

A crucial role of histopathological and immunohistochemical findings:

Immunohistochemical Evaluation:

• The sarcomatoid component demonstrated diffuse positivity for vimentin and smooth muscle actin (SMA), indicating mesenchymal differentiation. Notably, SATB2, although traditionally used as a marker in colorectal and osteoblastic tumors, has been observed in sarcomatoid elements, reflecting aberrant differentiation.

• The carcinomatous component retained epithelial characteristics, showing strong immunoreactivity for Pan-CK, CK7, and CK20.

• PSA was negative in both components, which is a known feature in dedifferentiated or high-grade variants and often correlates with more aggressive tumor biology.

These findings support the diagnosis of sarcomatoid carcinoma rather than a pure sarcoma or carcinosarcoma. The presence of epithelial markers in the carcinomatous portion and the biphasic morphology were key to reaching the final diagnosis. The integration of H&E features and IHC profiles was essential in ruling out:

• Primary prostatic sarcoma, which typically lacks epithelial markers like CKs or Pan-CK.

• STUMP, which is characterized by bland stromal proliferation and lacks significant mitotic activity or nuclear atypia.

• Carcinosarcoma, which may show divergent differentiation but usually has a known prior history of prostate cancer or therapy-related changes.

Hence, histopathology and IHC not only confirmed the biphasic nature of the tumor but also excluded other mimics, reinforcing their indispensable role in diagnosing such rare and aggressive neoplasms.

## Discussion

According to the WHO Classification (5th ed., 2022), sarcomatoid carcinoma of the prostate is categorized under prostatic epithelial tumors with biphasic morphology (4–6). The tumor comprises conventional carcinoma elements along with a malignant spindle cell (sarcomatoid) component, often mimicking high-grade sarcomas, carcinosarcomas, or mesenchymal tumors of the prostate.

The differential diagnosis on histopathology includes:

• Primary prostatic sarcoma

• Metastatic sarcomatoid carcinoma

• Carcinosarcoma

In this case, the diagnosis of sarcomatoid carcinoma of the prostate was strongly supported by both histopathological and immunohistochemical findings, while key differentials were systematically excluded. Primary prostatic sarcoma was ruled out due to the clear presence of a biphasic pattern - malignant spindle cells alongside distinct epithelial area, along with diffuse positivity for epithelial markers such as Pan-CK, CK7, and CK20, which are characteristically absent in pure sarcomas. Metastatic sarcomatoid carcinoma was considered; however, there was no clinical or radiological evidence of a primary malignancy elsewhere, and although site-specific markers like TTF-1 or GATA3 were not performed, the lack of any supportive systemic findings and the tumor’s immunophenotype aligned with a prostatic origin, not a metastasis. Carcinosarcoma was also excluded based on the absence of heterologous differentiation (e.g., osteoid, cartilage, or rhabdomyoblasts) and no history of prior prostate carcinoma or therapy, which are commonly associated with carcinosarcoma. Taken together, the biphasic morphology, immunoprofile, and clinical context make a compelling case for sarcomatoid carcinoma, while confidently ruling out its closest histological mimics.

The etiology of sarcomatoid carcinoma remains unclear. However, some studies suggest it may arise as a dedifferentiated variant of conventional prostate carcinoma, potentially driven by TP53 mutations, Rb1 loss, and epithelial-mesenchymal transition (EMT) (7,8). Due to its rarity, there are no established treatment guidelines, and management is often based on case reports and small retrospective studies (6,9). The prognosis of sarcomatoid carcinoma is generally poor, with a high likelihood of metastasis at presentation (2). Most patients exhibit resistance to androgen deprivation therapy (ADT), particularly those with PSA-negative tumors (5). Radical prostatectomy is an option for localized disease, but for advanced cases, a multimodal treatment approach is required, including chemotherapy and radiation therapy (9). Histopathologically, sarcomatoid carcinoma must be distinguished from other spindle cell neoplasms of the prostate, including stromal tumors and sarcomas. IHC markers such as vimentin and pan-cytokeratin are essential for accurate diagnosis (3,8). An epithelial component with glandular differentiation supports the diagnosis of sarcomatoid carcinoma rather than a primary sarcoma (7). The absence of PSA expression further suggests a loss of typical prostate epithelial characteristics (5). Regarding differential diagnosis, this tumor must be distinguished from other biphasic malignancies, including carcinosarcoma and prostatic stromal tumors of uncertain malignant potential (STUMP) (8). While carcinosarcoma also exhibits epithelial and sarcomatoid components, it is typically associated with a prior history of prostate cancer treatment, which was not the case in our patient (8,9). However, STUMP lacks the high-grade features and mitotic activity seen in sarcomatoid carcinoma (8).

## Conclusion

This case highlights the crucial role of meticulous histopathological and immunohistochemical evaluation in diagnosing and differentiating sarcomatoid carcinoma of the prostate from other spindle cell tumors. Early diagnosis and prompt treatment are essential given its rarity, aggressive nature, and overlapping histological features. Effective management requires a multidisciplinary approach involving urologists, oncologists, and pathologists to optimize patient outcomes. Further studies are needed to establish standardized treatment protocols and improve prognostic predictions, ultimately enhancing the understanding and management of this rare malignancy.

## Conflict of Interest

The authors have no conflict of interest.
